# To Clone or Not to Clone? Induced Pluripotent Stem Cells Can Be Generated in Bulk Culture

**DOI:** 10.1371/journal.pone.0065324

**Published:** 2013-05-29

**Authors:** Charlotte A. Willmann, Hatim Hemeda, Lisa A. Pieper, Michael Lenz, Jie Qin, Sylvia Joussen, Stephanie Sontag, Paul Wanek, Bernd Denecke, Herdit M. Schüler, Martin Zenke, Wolfgang Wagner

**Affiliations:** 1 Helmholtz-Institute for Biomedical Technology, Stem Cell Biology and Cellular Engineering, RWTH Aachen University Medical School, Aachen, Germany; 2 Aachen Institute for Advanced Study in Computational Engineering Science (AICES), RWTH Aachen University, Aachen, Germany; 3 Institute for Biomedical Engineering – Cell Biology, RWTH Aachen Medical School, Aachen, Germany; 4 Interdisciplinary Center for Clinical Research, RWTH Aachen Medical School, Aachen, Germany; 5 Institute of Human Genetics, RWTH Aachen Medical School, Aachen, Germany; University of Kansas Medical Center, United States of America

## Abstract

Induced pluripotent stem cells (iPSCs) are usually clonally derived. The selection of fully reprogrammed cells generally involves picking of individual colonies with morphology similar to embryonic stem cells (ESCs). Given that fully reprogrammed cells are highly proliferative and escape from cellular senescence, it is conceivable that they outgrow non-pluripotent and partially reprogrammed cells during culture expansion without the need of clonal selection. In this study, we have reprogrammed human dermal fibroblasts (HDFs) with episomal plasmid vectors. Colony frequency was higher and size was larger when using murine embryonic fibroblasts (MEFs) as stromal support instead of HDFs or human mesenchymal stromal cells (MSCs). We have then compared iPSCs which were either clonally derived by manual selection of a single colony, or derived from bulk-cultures of all initial colonies. After few passages their morphology, expression of pluripotency markers, and gene expression profiles did not reveal any significant differences. Furthermore, clonally-derived and bulk-cultured iPSCs revealed similar *in vitro* differentiation potential towards the three germ layers. Therefore, manual selection of individual colonies does not appear to be necessary for the generation of iPSCs – this is of relevance for standardization and automation of cell culture procedures.

## Introduction

Induced pluripotent stem cells open fascinating perspectives for drug discovery, cell therapy and basic research [1]. Reprogramming of somatic cells is generally achieved by ectopic expression of defined transcription factors. Various methods have been described, including transfection with episomal plasmid vectors which enable the generation of integration-free iPSCs [2]–[4]. Such integration-free iPSCs are of relevance for regenerative medicine since they diminish the risk of insertion-associated genetic aberrations [5], [6]. Initial colonies arise three to four weeks after induction and they typically reveal a heterogeneous morphology: pluripotent cells have relatively large nuclei and grow in flat colonies with an embryonic stem cell (ESC)-like morphology and with a sharp rim, whereas other colonies lack a sharp border and consist of larger and rather granular cells [7], [8]. It is commonly accepted that this heterogeneity of initial clones reflects either successful or partial reprogramming into iPSCs [9], [10].

In order to select for fully reprogrammed cells the colonies are usually scored by visual inspection of morphology [11]. Additionally, expression of pluripotency-associated glycoproteins on the cell surface can be assessed, such as stage-specific embryonic antigens 3 and 4 (SSEA3 and SSEA4) or tumor related antigens 1-60 or 1-81 (TRA-1-60 or TRA-1-81) [12]–[15]. Other enrichment strategies employ the introduction of antibiotic resistance or fluorescent proteins under the control of pluripotency-specific promoters [9], [16], [17]. To physically select iPS cells, suitable colonies are then picked up with a pipette and transferred to a new culture well for subsequent culture expansion [18] – the progeny is then derived from the same parental cell, and thus, clonally derived. This procedure is straight forward, but it is time-consuming and necessitates extensive training.

The selection of suitable colonies is however difficult to standardize as it is rather based on the experience of the operator than on objective criteria [15]. Furthermore, colony growth and morphology is dependent on cell culture conditions, such as the type of feeder cells. With regard to quality control of cell preparations - particularly in regenerative medicine - and with regard to automated high throughput processes, iPSC generation without the need of clonal selection would therefore be advantageous. One important feature of pluripotent cells is their escape from replicative senescence [19], [20]. Furthermore, these cells reveal high proliferation rates under appropriate culture conditions. It is therefore conceivable, that fully reprogrammed cells outgrow partially reprogrammed cells in the course of culture expansion.

In this study, we compared initial colony formation upon pluripotency induction on different types of feeder cells. These colonies were then either manually picked, or all colonies were harvested in bulk for subsequent expansion. After 10 passages colony morphology, proliferation rates, immunophenotype, gene expression profiles and *in vitro* differentiation potential did not show significant differences between clonally derived or bulk-cultured iPSCs.

## Materials and Methods

### Ethical statement

This study was approved by the Ethic Committee of the University of Aachen and all samples were isolated after written consent (MSC: permit number EK128/09; dermal fibroblasts: permit number 163/07).

### Cell culture

HDFs were isolated from skin samples of patients undergoing surgical interventions [21]. Briefly, skin samples were washed in phosphate buffered saline (PBS; PAA, Pasching, Austria) and treated with collagenase (Serva, Heidelberg, Germany) for 4 hours. After digestion, dermal remnants were removed by filtering the solution through a 100 µm nylon strainer (Falcon, Becton Dickinson, San Jose, USA). MSCs were isolated from the *caput femoris* of patients undergoing femoral head prosthesis [19], [22]. HDFs and MSCs were thoroughly characterized by a panel of immunophenotypic surface markers and *in vitro* differentiation towards adipogenic, osteogenic and chondrogenic lineages as described in our previous work [21]. MEFs were prepared as described elsewhere [23]. Cells were cultured at 37°C in a humidified atmosphere with 5% CO_2_ in culture medium consisting of Dulbecco’s Modified Eagles Medium (DMEM; PAA) with 2 mM L-glutamine (Sigma Aldrich, St-Louis, MO, USA) and 100 U/mL penicillin/streptomycin (Gibco), 10% fetal calf serum (FCS, PAA). Medium changes were performed twice per week and cells were passaged after reaching 80% confluency using a 0.25% trypsin-EDTA solution (Gibco) and counted with a Neubauer counting chamber (Brand, Wertheim, Germany).

### Preparation of irradiated feeder cells

For preparation of irradiated feeder layers, the cells were harvested, resuspended in culture medium at 1×10^6^ cells/ml, exposed to either 30 Gy (MSCs and MEFs) or 60 Gy (HDFs) using a blood product irradiator (model IBL-437, Foss Therapy Service, North Hollywood, CA, USA), and cryopreserved for subsequent application. For iPSC culture, feeder cells were seeded at a density of 23,000 cells/cm^2^.

### Reprogramming into iPS cells

iPSCs were generated using episomal plasmids as described previously [2]. In brief, HDFs at passage 2 or 3 were washed with PBS without Mg/Ca (PAA), and resuspended in 100 µl of R buffer (Life Technologies, Carlsbad, CA, USA) with either 3 µg of reprogramming plasmid mixtures (OCT3/4, siRNA for P53: pCXLE-hOCT3/4-shp53-F, accession no. 27077; SOX2, KLF4: pCXLE-hSK, accession no. 27078; L-MYC, LIN28: pCXLE-hUL, accession no. 27080; Addgene) or with 3 µg of a GFP control plasmid (pCXLE-EGFP, accession no. 27082; Addgene). The transfection was carried out with the NEON transfection system according to the manufacturer’s instructions (Life Technologies; 1,650 V, 10 ms, 3 time pulses). The cells were placed in one well of a 6-well plate containing 2 ml of pre-warmed medium (DMEM-LG with 2 mM L-glutamine and 10% FCS without antibiotics). The medium was then exchanged every second day using culture medium with 100 U/mL penicillin/streptomycin. 8 days after the transfection, cells were trypsinized and 1,700 cells/cm^2^ cells were plated in a 6-well plate either pre-seeded with irradiated feeder cells or without irradiated feeders as indicated in the text. The next day, culture medium was changed to hiPS medium consisting of Knockout-DMEM with 20% Knockout serum replacement, 100 U/ml penicillin/streptomycin, 2 mM l-Glutamin, 0.1 mM ß-Mercaptoethanol (all Gibco) and 10 ng/ml basic fibroblast growth factor (bFGF; PeproTech, NJ, USA). From then on medium was exchanged every day. Colony frequency was measured by counting emerging colonies at day 21–28 after transfection. Colony size was determined by measuring the colony area using Image J [24]. The calculated size of each individual colony was tracked over 4 days to estimate colony growth.

### Cloning and passaging of iPS cells

Manual picking of individual colonies was performed using an EVOS *fl* microscope (AMG, Bothell, WA, USA) under sterile conditions. Colonies were cut, harvested with a pipette and placed into new cell culture wells with feeder cells. For bulk culture experiments or passaging of established iPSC lines, all colonies in the well were dissociated using collagenase IV (Gibco) for 45 minutes. The collagenase was diluted by adding culture medium and the cells were rinsed gently. For bulk culture, cells were resuspended in 1 ml hiPS cell culture medium after centrifugation and 25 – 100 µl of cell suspension were seeded into new cell culture wells with feeder cells. Passaging was performed once or twice a week with collagenase as described above depending on cell density. The cells were then resuspended in hiPS cell culture medium and reseeded in 6-well-plates at a ratio of 1∶3–1∶6. To estimate proliferation, we have either analyzed the increase of colony-size as described above, or we performed direct cell counting. Therefore, equal amounts of cells were seeded at day 0 in different wells (6-well plates). The progeny of individual wells was then harvested after 1, 3 and 5 days using collagenase IV, separated in single cells with accutase treatment (Innovative Cell Technologies, San Diego, USA) and counted in a Neubauer counting chamber.

### Flow cytometry

To test the maintenance of episomal plasmids over time, we analyzed GFP-expression upon transfection using the reporter plasmid pCXLE-EGFP: with a size similar to the reprogramming plasmids. Once per week cells were fixed with 2% paraformaldehyde (PFA, Merck Millipore, Darmstadt, Germany), washed with PBS (PAA), and analyzed using a FACSCanto II flow cytometer (Beckton Dickinson, Heidelberg, Germany).

### Immunofluorescence staining

iPS cells or differentiated cells were seeded in 4-well plates (Nunc, Langenselbold, Germany) on Matrigel-(Beckton Dickinson, Heidelberg, Germany) or 0.1% gelatine (Sigma)-coated coverslips, respectively, cultured for 3 days and then fixed with 4% PFA. Following blocking of nonspecific binding with normal goat serum, cells were incubated overnight with the monoclonal primary antibody (Table S1 in File S1) in PBS at 4 °C. If indicated, double staining for OCT4 was performed in PBS/0.1% Triton-X-100 (Sigma). After three washing steps with PBS, cells were stained in the dark for 1 hour with the secondary IgM or IgG antibody conjugated with either Alexa594 or FITC (Table S1 in File S1). After three washing steps, counterstaining with DAPI (4′,6-Diamidin-2-phenylindol, Sigma Aldrich) was performed, and the slips were covered on slides with mounting solution (Dako, Glostrup, Denmark). For each experiment we analyzed at least three biological replicas (>10 individual colonies per experiment). Images were acquired using an Axioplan 2 fluorescence microscope (Carl Zeiss, Oberkochen, Germany).

### *In vitro* differentiation

Differentiation of iPSC lines was evaluated using the embryoid body (EB) assay. In brief, iPS cells were enzymatically detached and placed into ultra-low-attachment cell culture plates (Corning, Corning, NY, USA) containing differentiation medium (hiPS medium with 20% FCS, without KO Serum Replacement and without bFGF). After one week, the EBs were transferred to gelatine-coated 12-well plates and allowed to attach for 10 days. Cells were harvested at day 0, 7, 13 and 17 of differentiation for RNA isolation. Immunofluorescent staining and microscopic analysis was performed at day 17 as described above using a BZ 9000 fluorescence microscope (Keyence, Neu-Isenburg, Germany).

### Quantitative RT-PCR

For the analysis of genomic integration of episomal plasmids, DNA was isolated using the QIAamp DNA Blood Mini Kit (Qiagen, Hilden, Germany). For the differentiation assay, RNA was isolated using the NucleoSpin RNA II Isolation Kit (Macherey Nagel, Düren, Germany) and cDNA was generated using the High Capacity cDNA Reverse Transcription Kit (Applied Biosystems, Carlsbad, CA, USA). The PCR was performed using Applied Biosystems StepOnePlus device with the TaqMan Power SYBRGreen Master Mix (Applied Biosystems) and appropriate primers for exo-and endogenous pluripotency markers or several lineage markers, respectively (Table S2 in File S1). The relative expression level of the reference gene GAPDH was used for normalization. The efficiency of PCR (slope of Ct values) was very similar with all different primer pairs. To estimate the number of episomal plasmids, we could therefore use the ratio of plasmid derived sequences to endogenous sequences (two copies per cell).

### Cytogenetic analysis

Normal chromosomal constitution was verified by conventional karyotyping of the cultured iPSCs. Metaphase spreads were prepared using standard procedures of blocking cell division at metaphase, hypotonic treatment, and methanol/acetic acid fixation (3∶1). The staining and banding techniques included the use of the fluorescence dye quinacrine (QFQ-banding) and a trypsin pretreatment (GTG-banding) carried out at 300 to 400 band level according to standard protocols. Microscopy was performed with Axioplan fluorescence microscope (Carl Zeiss, Jena, Germany) and IKARUS^™^ and ISIS^™^ digital imaging systems (MetaSystems, Altlussheim, Germany). Eight to 20 QFQ and GTG banded metaphases were analyzed per sample.

### PluriTest/Microarray analysis

RNA was isolated from iPSCs at passage 10 using a NucleoSpin miRNA kit (Macherey Nagel, Düren, Germany) and the RNA quality was evaluated by using a BioAnalyzer (Agilent, Böblingen, Germany). Gene expression profiles were then analyzed using GeneChip Human Gene 1.0 ST microarrays (Affymetrix, Santa Clara, CA, USA) according to the manufacturer’s instructions. All data are accessible at NCBIs Gene Expression Omnibus (http://www.ncbi.nlm.nih.gov/geo/query/acc.cgi?token=pnizxmsiasygctu&acc=GSE42807). Data were preprocessed with the Robust Multichip Average (RMA) method using apt-probeset-summarize from the Affymetrix Power tools software suite (http://www.affymetrix.com/partners_programs/programs/developer/tools/powertools.affx) and further analyzed using the Multi Experiment Viewer (MeV, part of TM4 Microarray Software Suite) [25]. Hierarchical clustering was performed with Pearson correlation. Significance analysis for microarrays (SAM) was used to search for differences between bulk and clonally-derived iPSCs [26]. For evaluation of pluripotency based on gene expression profiles we applied the PluriTest [27] with a reference dataset consisting of 98 pluripotent and 1,028 non-pluripotent samples as described before [28]. As a reference we have used a previously published dataset on gene expression profiles of human fibroblasts, ESCs and iPSCs which were analyzed on the same platform (GSE21655) [29]. These data were quantile normalized together with our data and a selection of pluripotency markers was used for heatmap presentation.

### Statistics

Results are expressed as mean ± standard error of at least three independent experiments. For significance assessment, we used the two-sided Student’s T-test.

## Results

### Colony morphology on different feeder layers

**Figure 1:**
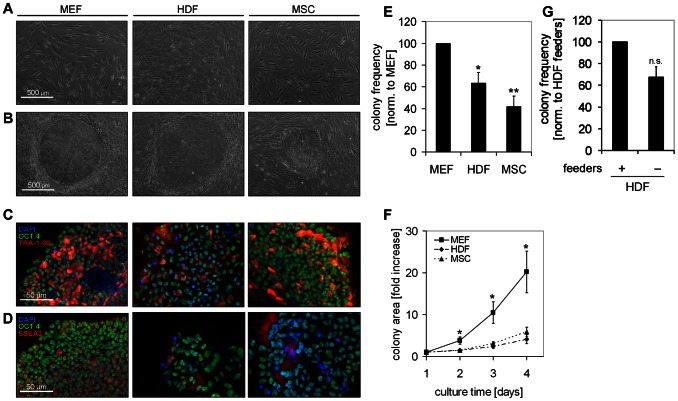
iPSC colonies on different feeder layers. Three types of irradiated feeder layers have been used: murine embryonic fibroblasts (MEFs), human dermal fibroblasts (HDFs), and human bone marrow derived mesenchymal stromal cell (MSCs) (**A**). The morphology of iPSC colonies is exemplarily presented on each of these feeder layers (**B**). iPSC colonies were further characterized by immunofluorescent staining for the pluripotency markers OCT4 (green), TRA-1-60 (red) and SSEA3 (red) with nuclear counterstaining (DAPI, blue) (**C,D**). The initial colony-frequency and increase of colony-size was compared on different feeder layers: colony-frequency was significantly higher on irradiated MEFs than on HDFs (P = 0.03), or MSCs (P = 0.01; total colony counts (in four experiments): 333 with MEF, 225 with HDF, and 153 with MSC) (**E**) and the colony-size increased faster on MEFs than on HDFs or MSCs (P = 0.01 and P = 0.13, respectively) (**F**). Alternatively, induced fibroblasts were re-seeded in wells without preformed irradiated feeder cells. Non-induced fibroblasts from the parental cell preparation then grew out to provide a stromal support for iPSC colonies but their frequency was lower than using preformed irradiated feeders (total colony counts (in three experiments): 215 with HDF and 132 without feeder) (**G**). (** = P<0.01; * = P<0.05; n.s. =  not significant).

Maintenance of pluripotency in culture is supported by stromal feeder cells [13]. We have reprogrammed human dermal fibroblasts using episomal plasmids. After seven days the cells were reseeded in parallel on three different feeder layers: irradiated murine embryonic fibroblasts (MEFs), irradiated human dermal fibroblasts (HDFs), and irradiated human bone marrow derived mesenchymal stromal cells (MSCs; Figure 1A). After three weeks, colonies formed on each of these feeders: their morphology was similar although the appearance - particularly the rim of the colonies – is affected by the underlying stromal layer. This exemplifies the difficulties to categorize colonies by visual inspection (Figure 1B). Colonies were further analyzed by immunofluorescent staining for the pluripotency markers POU5F1 (OCT4), TRA-1-60 and SSEA3 demonstrating that MEFs, HDFs, and MSCs support formation of typical ESC-like colonies (Figure 1C,D). Comparison of the support of three types of feeders revealed that colony-frequency was significantly higher on MEFs than on HDFs (P = 0.03), or MSCs (P = 0.01; Figure 1E). Furthermore, the colony-size increased faster on MEFs than on HDFs or MSCs (Figure 1F). These differences in stromal support may be due to the different species, onthology or cell types. Either way, the results support the notion that human fibroblasts and MSCs provide suitable alternatives if xeno-free culture conditions are required [30].

Alternatively, we seeded the transfected fibroblasts without stromal support: then, the non-induced fibroblasts from the parental cell preparation grew out to support initial colony formation. However, the colony-frequency was lower than using irradiated feeders and after one passage proliferation of fibroblasts was not fast enough to sustain pluripotent colonies (Figure 1G). These results indicate that parental cells can be used as initial feeder layer. Nevertheless, MEFs provided the best stromal support – whether cell type or species dependent – and they were therefore used for subsequent experiments.

### Generation of iPSC-colonies in bulk culture

**Figure 2:**
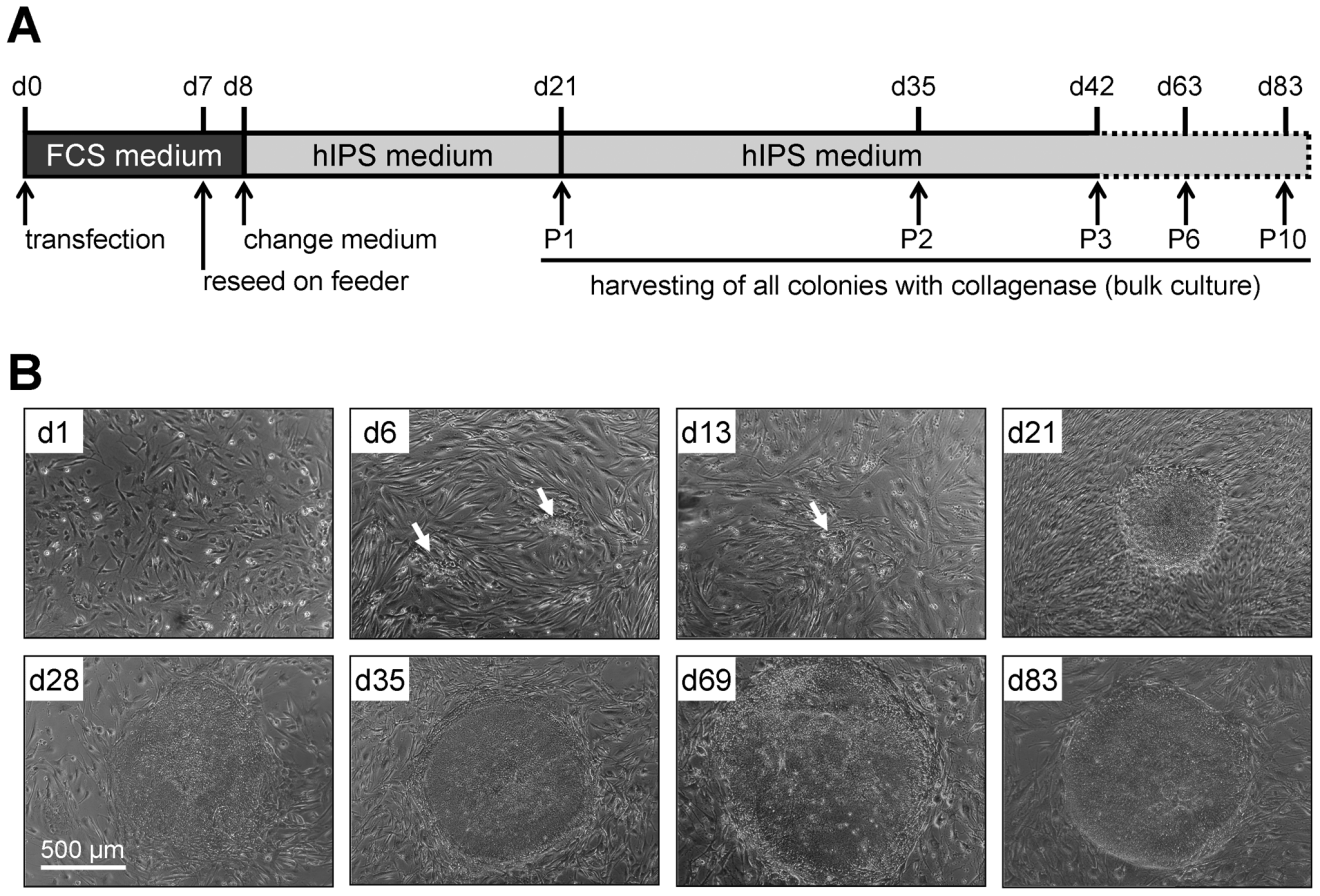
Generation of iPSCs in bulk culture. Time axis for reprogramming of human dermal fibroblasts with episomal plasmids (**A**). Exemplary phase contrast images of bulk-cultured colonies are depicted in the course of culture-expansion. Already at day 6 the first changes in cell morphology could be noticed (arrows). Bulk cultured colonies revealed a typical ESC-like morphology after 10 passages (**B**).

**Figure 3:**
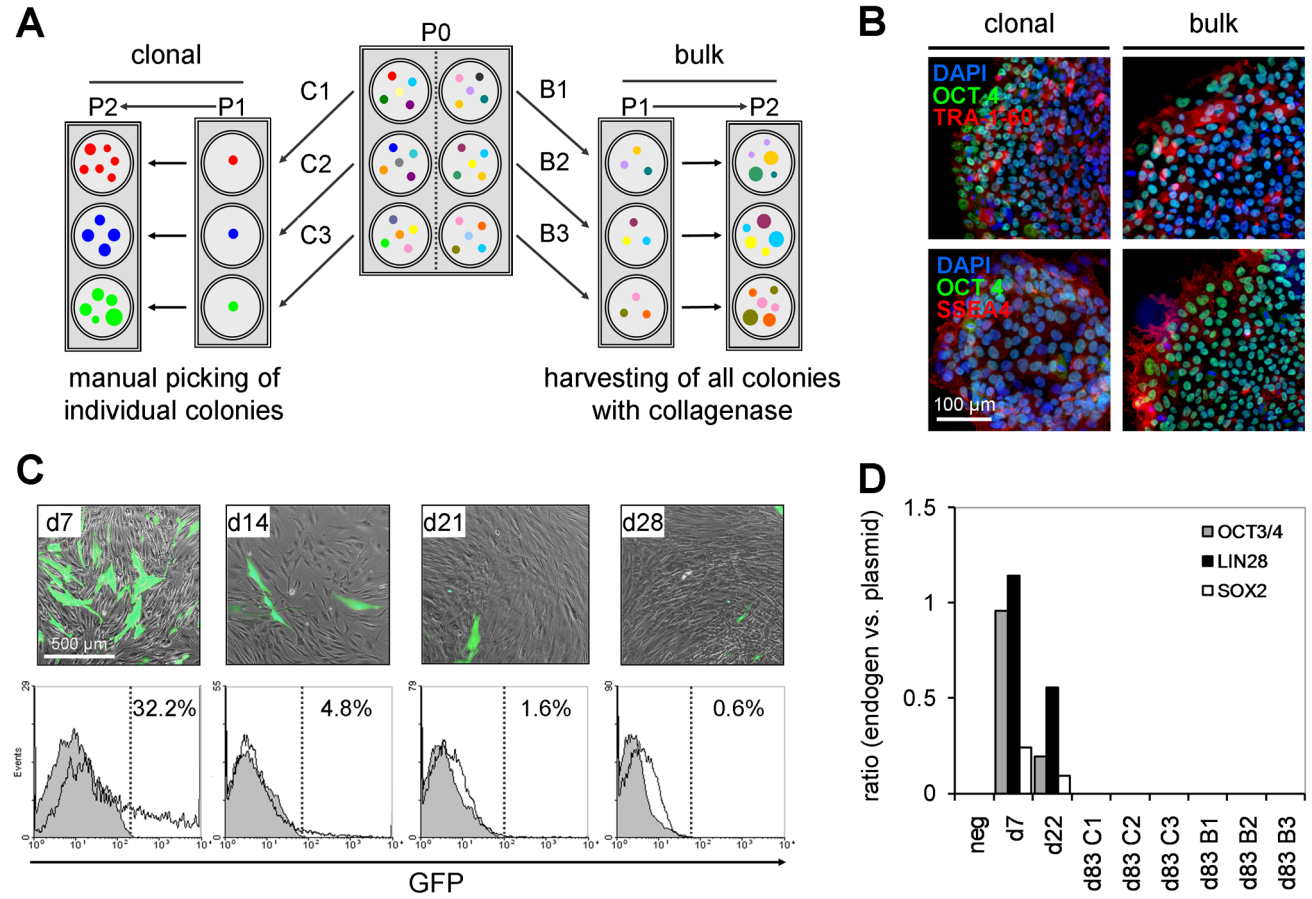
Comparison of cloned and bulk-cultured iPSC colonies. Three weeks after transfection, individual ESC-like colonies were either manually picked from culture wells corresponding to independent transfections (to ensure clonal derivation from different parental cells), or all colonies per well were dissociated using collagenase IV and re-seeded on new feeder layer (bulk-culture) (**A**). Comparison of clonal and bulk-cultured iPSCs at passage 10 did not reveal any differences in immunofluorescence staining for OCT4 (green), TRA-1-60 and SSEA4 (red) with DAPI counterstaining (blue) (**B**). Loss of episomal plasmids over time is exemplified in fibroblasts which were transfected with an episomal vector for green fluorescent protein (GFP; representative results of three independent experiments are demonstrated) (**C**). Quantitative PCR analysis of the reprogramming factors (OCT3/4, LIN28, and SOX4) encoded by the three episomal vectors and their endogenous counterparts did not reveal stable integration in clonal and bulk-cultured colonies: episomal plasmids were only detected at day 7 and day 22 after transfection but not in any of the established iPSCs at passage 10 (Representative results of three independent experiments are demonstrated) (**D**).

To analyze if culture isolation of iPSCs is feasible without clonal selection we have harvested all initial colonies by collagenase treatment and reseeded them on MEFs. Over the passages, colony morphology became more and more homogenous indicating that successfully reprogrammed colonies outgrew the partially differentiated colonies (Figure 2). We have then systematically compared bulk-cultured iPSCs with clonally derived iPSC lines. Induction of pluripotency was performed six times in parallel: three of these preparations were used to pick individual colonies after 21 days based on their morphology; the other three wells were harvested by collagenase treatment for further expansion as bulk culture (Figure 3A). After expansion for 10 passages analysis of pluripotency markers revealed some variation between individual colonies, which might be attributed to heterogeneity within iPSCs or to the staining procedure and which renders a direct comparison of expression level difficult. Overall, comparison of clonally-derived or bulk-cultured iPSCs did not reveal differences in expression of OCT4, TRA-1-60 and SSEA4 (Figure 3B). Clonally-derived and bulk-cultured colonies revealed similar morphology (Figure S1 in File S1). Furthermore, they were passaged at the same time intervals indicating that their proliferation rate was similar and this was validated by analysis of colony growth and cell counting (Figure S2 in File S1).

### No integration of episomal plasmids or karyotypic abnormalities

Usually, episomal vectors are lost from culture within several weeks as exemplified by an episomal plasmid encoding the green fluorescent protein (GFP; Figure 3C; Figure S3 in File S1). However, occasional genomic integrations have been described [31]. Such integrations might occur particularly during the transfection procedure and therefore, we anticipated that bulk-cultured cells - which resemble a mixture of initial colonies - might be more prone to genomic integration. We used quantitative PCR analysis to detect reprogramming factors encoded by the three episomal vectors and their endogenous counterpart. Episomal plasmids were only detected at day 7 and day 22 of clonal and bulk-culture after transfection but not in any of the established iPSCs at passage 10 (Figure 3D). Furthermore, we have performed karyotypic analysis of clonally-derived and bulk-cultured iPSCs and we did not observe chromosomal abnormalities (Figure S4 in File S1).

### Gene expression profiles of clonal and bulk-cultured iPSCs

**Figure 4:**
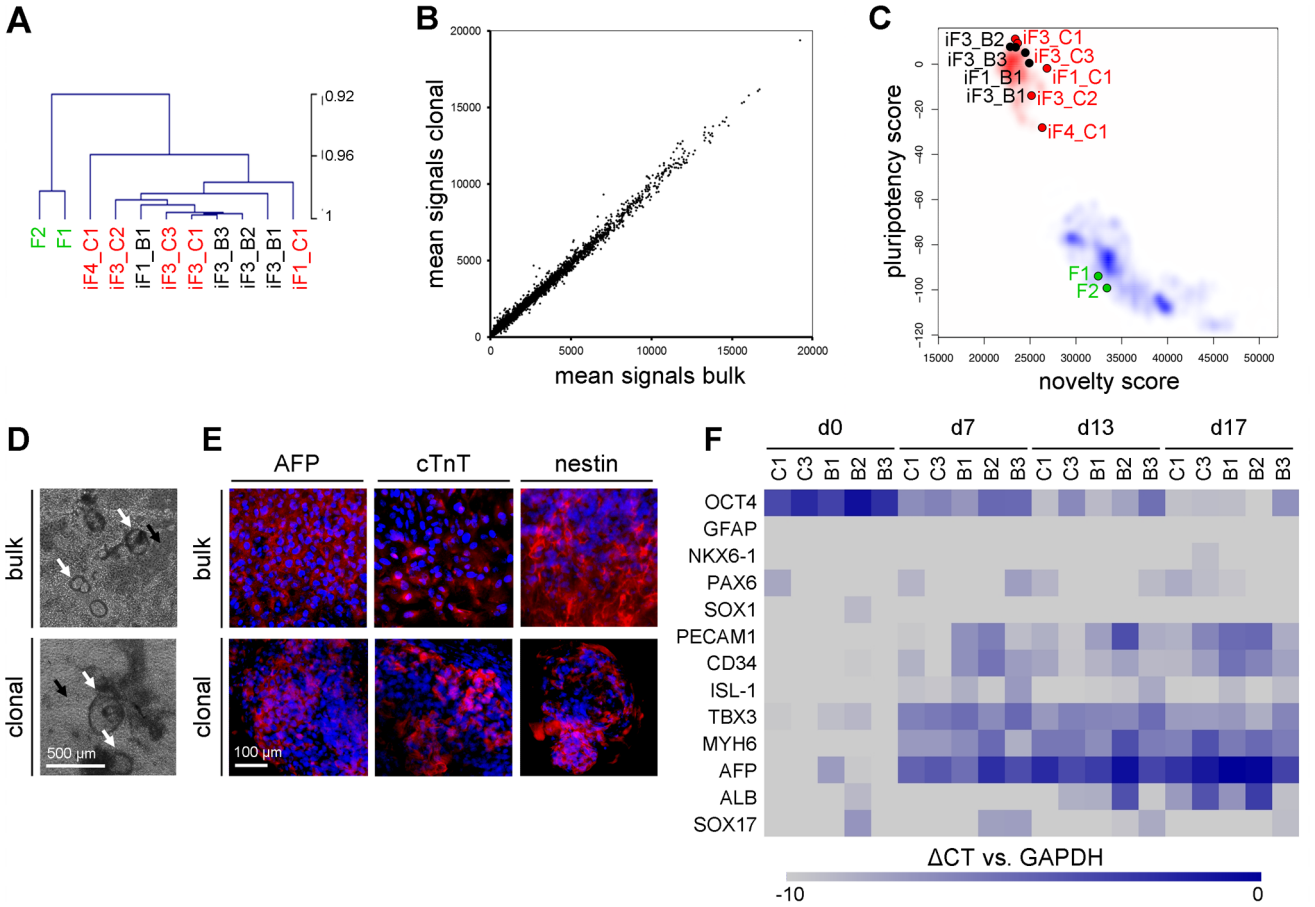
Gene expression profiles and *in vitro* differentiation of iPSCs. Hierarchical clustering of global gene expression profiles revealed clear separation of non-transfected fibroblasts (F1, F2; green) and iPSCs (bulk: black; clonal: red). iF2_B1 and iF2_C1 grew nicely until passage 3, but were then lost due to bacterial contamination. iF4 was only cultured clonally without bulk counterpart. (**A**). Scatterplot analysis of mean signal intensity demonstrated very similar gene expression profiles of clonal and bulk-cultured iPSCs and statistical analysis did not reveal significant differences (**B**). PluriTest analysis supported the notion that bulk-cultured as well as clonally-derived iPSCs are pluripotent as they grouped with established pluripotent cells (red area) and not with somatic samples (blue area) **(C)**. *In vitro* differentiation potential of iPSC colonies was evaluated by an embryoid body (EB) assay. After 10 days, each cell preparation revealed cystic structures (black arrows), areas with cells of epithelial morphology (white arrows) and spontaneous beating areas indicating cardiac differentiation (**D**). Up-regulation of differentiation markers AFP (endodermal marker), cTnT (mesodermal marker) and nestin (ectodermal marker) could be induced in all bulk-cultured and clonally derived iPSCs (**E**). Expression of ectodermal (GFAP, NKX6-1, PAX6, SOX1), mesodermal (PECAM1, CD34, MYH6), and endodermal markers (AFP, ALB, SOX17) was assessed by RT-qPCR: all of these genes were up-regulated upon differentiation, whereas OCT4 expression was down-regulated (**F**).

Subsequently, we compared gene expression profiles of induced fibroblasts (“iF”) from three different donors which were either clonally derived (“C”) or bulk-cultured (“B”). Hierarchical clustering revealed clear separation of fibroblasts and iPSCs (Figure 4A). Scatterplot analysis of mean signal intensity demonstrated very similar gene expression profiles of clonally derived *versus* bulk-cultured iPSCs (Figure 4B). Significance analysis of microarray (SAM) did not reveal any significant gene expression changes indicating that the initial clonal selection did not exert reproducible effects on cell preparations. Gene expression level of various pluripotency genes was directly compared to previously published data on ESC and iPSC and the results were very similar [29] (Figure S5 in File S1). Furthermore, gene expression profiles were analyzed using PluriTest which is regarded as a sensitive and highly specific, animal-free alternative to teratoma assays for assessing the pluripotency [27], [28]. All bulk-cultured and clonally-derived iPSCs grouped together with established pluripotent stem cell lines and were clearly separated from somatic samples. Only the clonally derived iPSC preparation from donor 4 appeared to be partially differentiated (Figure 4C).

### *In vitro* differentiation using the embryoid body assay

Bulk-cultured and clonally-derived iPSCs were capable of embryoid-body (EB) formation and these spheroids were plated on gelatin coated tissue culture plastic after seven days for further differentiation. After 10 days each cell preparation revealed cystic structures, areas with epithelial morphology, and spontaneous beating areas indicating cardiac differentiation (Figure 4D; Video S1 in File S2). Expression of alpha-fetoprotein (AFP; endodermal marker), cardiac troponin T (cTNT; mesodermal marker) and nestin (NES; ectodermal marker) were observed by immunofluorescent microscopy in each differentiated cell preparation (Figure 4E). Furthermore, differentiation towards all three germ layers was analyzed by RT-qPCR: several differentiation markers for the three germ layers were up-regulated, whereas OCT4 expression was down-regulated (Figure 4F; Figure S6 in File S1). These results support the notion that clonally-derived as well as bulk-cultured cells resemble fully reprogrammed iPSCs.

## Discussion

Pluripotent cells have unlimited self-renewal potential – they can virtually be passaged infinitely without any signs of replicative senescence [32]. In contrast, somatic cells – and the differentiated progeny of pluripotent cells - enter a senescent state after a limited number of cell divisions [19], [20], [33]. It may therefore be anticipated, that long-term culture selects for fully reprogrammed iPSCs without requirement of any clonal selection by the operator. Recently, it has been suggested that enrichment of iPSCs after reprogramming can be assisted by positive selection of CD326 (EpCAM) positive cells [34] – this approach would also result in polyclonal iPSCs. In this study, we demonstrate that fully reprogrammed iPSCs can be generated in bulk-culture without clear differences in gene expression profiles or differentiation potential as compared to their clonally derived counterparts – however, the question remains if picking of individual clones is advantageous for iPSC generation.

It has been suggested that cloning of iPSC-lines is necessary for homogenous iPSC-cultures [9] – but this assumption is difficult to validate as there are notoriously differences between individual iPSC lines: the quality of iPSCs is greatly affected by culture conditions and changes over passages [35]. Furthermore, there is even heterogeneity within individual colonies [15], [36], [37]. This variation is also reflected in our immunofluorescence analysis of OCT4, TRA-1-60 and SSEA4. However, quantification of overall gene expression of pluripotency markers and comparison of differentiation potential did not reflect significant differences between clonally- and bulk-derived iPSCs. Specific iPSC lines are biased towards specific lineages [38], [39]: for example, our HDF-derived iPSCs appear to be biased towards the mesodermal lineage in comparison to iLB c1-30m-r12 iPSCs [40], which revealed particularly differentiation towards ectodermal lineage (Figure S7 in File S1). On the other hand, continuous passaging of iPSC further diminishes differences in DNA methylation between iPSC and ESC [35]. Therefore, differences which may exist due to the starting material – or which arise between individual colonies – may be nivelated during long-term expansion [41].

iPSC lines, as well as ESCs, reveal differences in their genetic and epigenetic profiles [42]. We have recently compared global DNA-methylation profiles of iPSC clones which were generated from human bone marrow derived MSCs [28]. Notably, those iPSC lines which were derived from the same donor clustered always closely together. This was somewhat unexpected as MSCs resemble very heterogeneous cell preparations [43] and therefore, their reprogrammed progeny might also resemble considerable differences. On the other hand, reprogramming had relatively little impact on DNA methylation at those CpG sites which reveal high variation between different donors indicating that iPSCs maintain some donor-derived epigenetic differences [28]. The results of this study support the notion that iPSC lines derived from the same donor are closely related – whether they are clonally derived or not.

Clonally derived iPSCs may harbor fewer mutations. Obviously, reprogramming with retroviral or lentiviral vectors might entail a potpourri of integration sites if iPSCs were polyclonally derived - this would increase the risk of insertion related mutagenesis. We did not detect any genomic integration of episomal reprogramming factors, but a larger number of iPSC lines needs to be analyzed to determine if reprogramming-associated mutations are enriched in polyclonally derived iPSCs [44]. On the other hand, it has been shown that individual clones “capture” the mutational history of their parental cell [45]. Fibroblasts may already acquire genomic aberrations during initial cell-isolation. Such mutations would only be detected by karyotypic analysis or single nucleotide polymorphism (SNP) arrays if they entailed a significant growth advantage *in vitro* [46], [47]. We did not observe chromosomal abnormalities in our cell preparations – neither in clonally-derived, nor in bulk-cultured iPSCs. In our previous work we have performed karyotypic analysis of MSCs at early and late passage and did not observe karyotypic differences, too [46]. However, other authors demonstrated transient aneuploidy of MSC without malignant transformation [48]. It is therefore well conceivable that clonally derived iPSCs are more homogenous and this should be systematically addressed in a larger number of cell preparations – preferentially by deep sequencing technology. With regard to mutations in the starting population it may therefore be advantageous to use single clone derived iPSCs.

There has been a lot of research to optimize reprogramming procedures, whereas the parameters for clonal selection and the molecular features of partially-reprogrammed cells have been less addressed. Selection of colonies for picking and culture expansion is largely dependent on the operator [49]. If this selection would have impact on the established cell lines then this procedure would need to be more standardized. There have been attempts to use algorithm-based image analysis based on colony size, shape, density and texture of the colony-rim. This analysis needs to take specific culture conditions, such as different types of feeder cells, into account. It is not trivial to standardize cell culture procedures but this is a prerequisite for reliable clinical applications.

Automation of cell culture and high-throughput generation of iPSCs provides fascinating perspectives for drug-screening and analysis of patient-specific iPSCs. For example, in our StemCellFactory consortium we are aiming for optimized protocols to automate generation, culture expansion and differentiation of iPSCs (www.stemcellfactory.de). Identification of suitable clones and colony picking are difficult to automate although devices, such as the CellCelector [50], [51], have been developed to facilitate image-based identification of colonies and picking. This may facilitate better standardization of clonal selection but automated cell cloning systems are relatively costly and failure-prone. Our approach may provide an easier and less costly alternative as it does not require automated identification and picking of successfully reprogrammed colonies.

## Conclusion

Selection and cloning of suitable colonies resembles a dogma in iPSC research – yet, it is unclear if cloning assures more homogeneous pluripotent cells. We demonstrate that generation of fully reprogrammed iPSCs is feasible in bulk culture – and, notably, the generated iPSC lines are indistinguishable from conventional clonally derived iPSC lines with regard to gene expression profiles and differentiation potential. More samples are necessary to unequivocally demonstrate if clonally- and bulk-cultured iPSCs are really alike in their differentiation potential and there may be more somatic mutations in bulk-cultured iPSCs. Furthermore, it is yet unclear, if bulk-cultured cells are really polyclonal, or whether one reprogrammed subclone outgrowths the rest. Either way, our data demonstrate that iPSC generation can be performed without clonal selection. Probably, partially-reprogrammed or differentiated cells are bound to senescence and disappear from culture after serial passages. The use of bulk-cultured iPSCs provides new perspectives for automated iPSC applications and may facilitate better standardization than experimenter driven selection of suitable clones.

## Supporting Information

File S1 **Combined supporting information file of additional figures and tables.** Figure S1. Morphology of clonally-derived and bulk-cultured iPSC colonies. Figure S2. Proliferation rate of clonally-derived and bulk-cultured iPSCs. Figure S3. FACS analysis of cells with episomal GFP expression. Figure S4. Conventional karyotyping of bulk-cultured iPSCs. Figure S5. Heatmap of pluripotency genes. Figure S6. Gene expression upon in vitro differentiation of iPSCs. Figure S7. In vitro differentiation of iLB c1-30m-r12 iPSCs. Table S1. Antibodies used in this study. Table S2. Primer sets used in this study.
(PDF)

File S2 **Video of spontaneous beating areas in bulk-cultured iPSCs.**
(WMV)
