# Supplementary material for: To Clone or Not to Clone? Induced Pluripotent Stem Cells Can Be Generated in Bulk Culture
Source: PLoS One. 2013 May 29;8(5):e65324. doi: 10.1371/journal.pone.0065324 (PMC3667031; doi:10.1371/journal.pone.0065324)
Supplement: File S1 — Combined supporting information file of additional figures and tables. Figure S1. Morphology of clonally-derived and bulk-cultured iPSC colonies. Figure S2. Proliferation rate of clonally-derived and bulk-cultured iPSCs. Figure S3. FACS analysis of cells with episomal GFP expression. Figure S4. Conventional karyotyping of bulk-cultured iPSCs. Figure S5. Heatmap of pluripotency genes. Figure S6. Gene expression upon in vitro differentiation of iPSCs. Figure S7. In vitro differentiation of iLB c1-30m-r12 iPSCs. Table S1. Antibodies used in this study. Table S2. Primer sets used in this study. (PDF) [file pone.0065324.s001.pdf]

Supplemental Material

To clone or not to clone? Induced pluripotent stem cells can be generated in bulk culture

Charlotte A. Willmann, Hatim Hemeda, Lisa A. Pieper, Michael Lenz, Jie Qin, Sylvia Joussen, Stephanie Sontag, Paul Wanek , Bernd Denecke, Herdit M. Schüler, Martin Zenke, Wolfgang Wagner

Index

Supplemental figure 1. Morphology of clonally-derived and bulk-cultured iPSC colonies. 1
Supplemental figure 2. Proliferation rate of clonally-derived and bulk-cultured iPSCs. 2
Supplemental figure 3. FACS analysis of cells with episomal GFP expression. 2
Supplemental figure 4. Conventional karyotyping of bulk-cultured iPSCs. 3
Supplemental figure 5. Heatmap of pluripotency genes. 4
Supplemental figure 6. Gene expression upon *in vitro* differentiation of iPSCs. 5
Supplemental figure 7. *In vitro* differentiation of iLB c1-30m-r12 iPSCs. 6
Supplemental Table 1. Antibodies used in this study. 7
Supplemental Table 2. Primer sets used in this study. 8

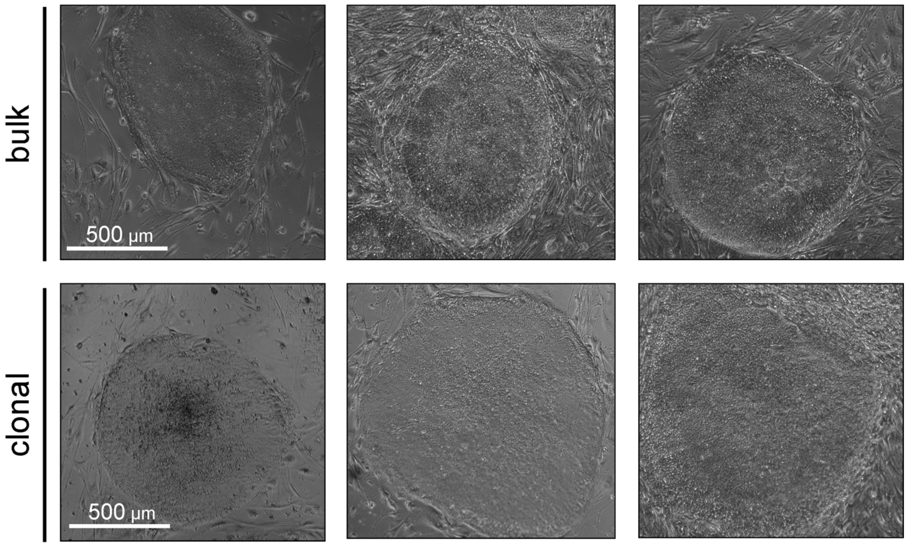

Supplemental figure 1. Morphology of clonally-derived and bulk-cultured iPSC colonies.

Clonal and bulk-cultured iPSCs at passage 10 revealed similar morphology. These representative images correspond to B1, B2, B3 and to C1, C2, and C3 respectively.

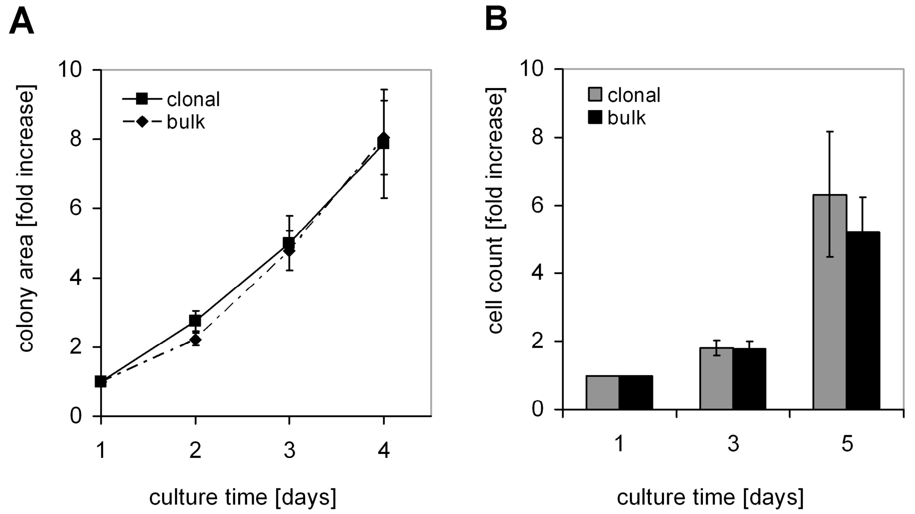

Supplemental figure 2. Proliferation rate of clonally-derived and bulk-cultured iPSCs.

(A) Proliferation of three clonally-derived and two bulk-cultured iPSCs was estimated by increased colony size over four days (four technical replicas per iPSC). **(B)** Alternatively, equal amounts of cells were seeded in individual wells and counted after 1, 3, and 5 days. Fold-increase in cell counts is depicted in comparison to day 1. The results demonstrate that bulk-cultured and clonally-derived iPSCs have similar proliferation rates.

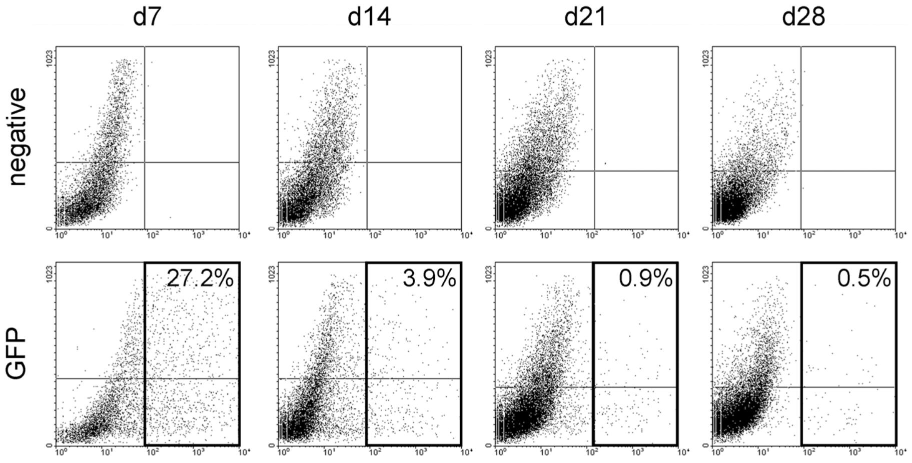

Supplemental figure 3. FACS analysis of cells with episomal GFP expression.

Dot plot analysis demonstrates GFP-expression *versus* forward scatter in cells transfected with an episomal plasmid for GFP or negative controls. The percentage of GFP-positive cells decays over four weeks. Representative data for three independent experiments are demonstrated.

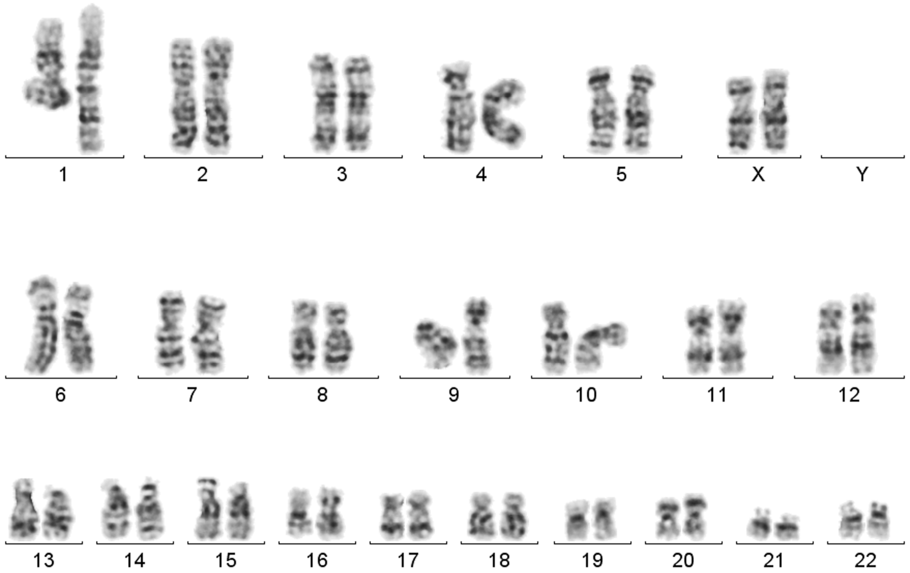

Supplemental figure 4. Conventional karyotyping of bulk-cultured iPSCs.

To investigate structural and numerical chromosomal alterations we performed conventional karyotyping on QFQ- and GTG-banded metaphases of clonally-derived or bulk-cultured iPSCs. A GTG-banded metaphase of a bulk-cultured iPSC preparation is exemplarily presented. We did not observe karyotypic abnormalities.

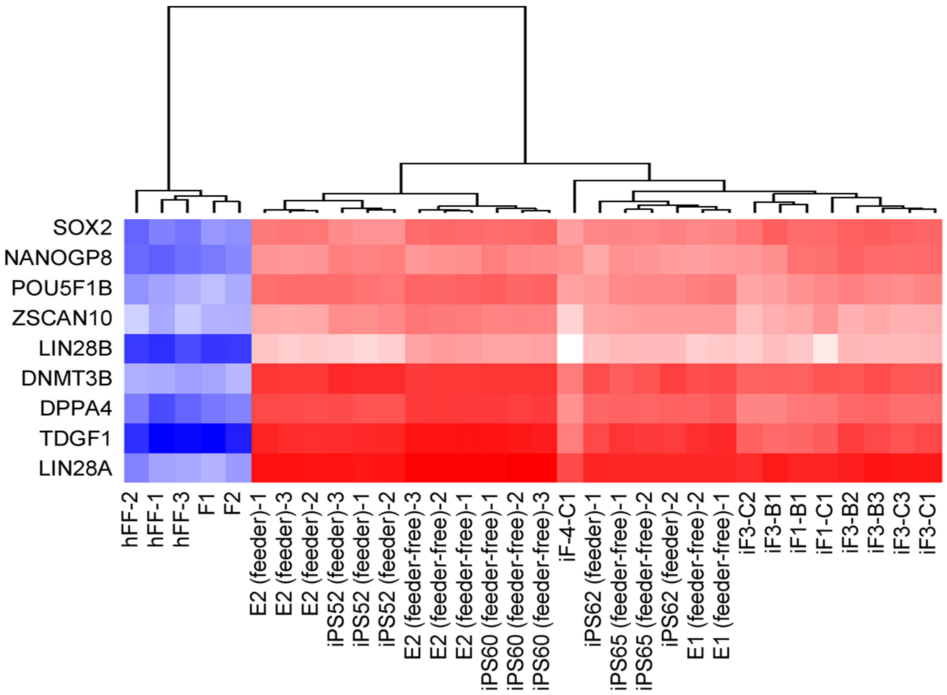

Supplemental figure 5. Heatmap of pluripotency genes.

Gene expression of selected pluripotency genes in our data was compared to previously published data on human fetal fibroblasts (hFF1/2/3), ESCs (E1/2) and iPSCs (iPS52, iPS60, iP62 and iPS65; GSE21655). This heatmap demonstrates very similar expression of pluripotency genes in the two different datasets (whereas they are not expressed in the corresponding fibroblasts).

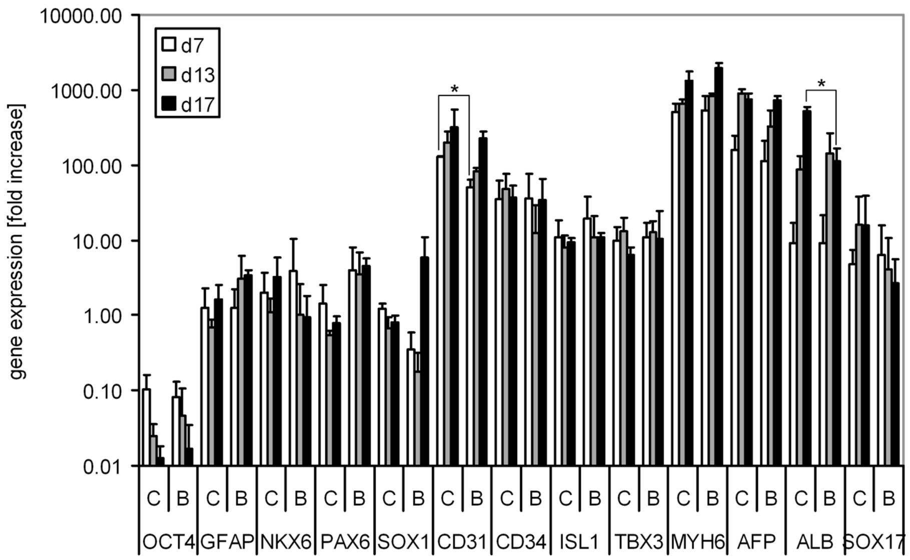

Supplemental figure 6. Gene expression upon *in vitro* differentiation of iPSCs.

Clonally-derived ("C") and bulk-cultured ("B") iPSCs were differentiated with embryoid-body formation and subsequently cultured on gelatin coated plastic for 7, 13, and 17 days. Differential expression in comparison to the corresponding non-differentiated iPSCs revealed up-regulation of ectodermal (GFAP, NKX6-1, PAX6, SOX1), mesodermal (CD31, CD34, ISL1, TBX3, MYH6), and endodermal markers (AFP, ALB, SOX17). In contrast, OCT4 expression was down-regulated. These data were assessed by RT-qPCR in three biological replica for bulk-cultured iPSCs and two biological replica (with an additional technical replica) for clonally derived iPSCs. Expression of PECAM1 (CD31) and albumin (ALB) may indicate differences between C and B. However, significantly different fold changes were only observed at individual time points and they are probably due to technical noise. Overall, up-regulation of differentiation markers was very similar in clonally-derived and bulk-cultured cells (* = $P < 0.01$).

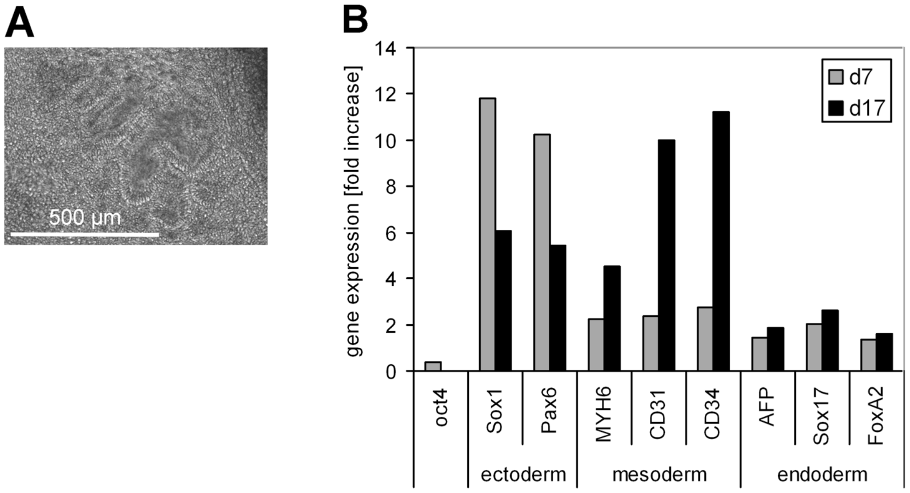

Supplemental figure 7. *In vitro* differentiation of iLB c1-30m-r12 iPSCs.

(A) Neuroectodermal rosettes were frequently observed upon differentiation of iLB c1-30m-r12 iPSCs. **(B)** Expression levels of the pluripotency marker OCT4 and ectodermal (PAX6, SOX1), mesodermal (MYH6, CD31, CD34), and endodermal markers (AFP, SOX17, FoxA2). These results were analyzed by RT-qPCR in analogy to supplemental figure 4.

Supplemental Table 1. Antibodies used in this study.

Antibody (label)	Clone	Source
Tra-1-60	TRA-1-60	Millipore
Tra-1-81	TRA-1-81	Stemgent
SSEA-3	MC-631	Millipore
SSEA-4	MC-813-70	Millipore
Oct4	C-10	Santa Cruz
AFP	189502	Millipore
cTnT	200805	R&D
Nestin	10C2	R&D
Goat α-mouse IgM (Alexa594)	A-21044	Molecular Probes
Goat α-rat IgM (Alexa594)	A-21213	Molecular Probes
Goat α-mouse IgG (Alexa594)	A-11005	Molecular Probes
Goat α-mouse IgG (FITC)	F-11021	Molecular Probes
Goat α-rabbit IgG (FITC)	F9887	Sigma

Supplemental Table 2. Primer sets used in this study.

Target		Primer sequence: Sense	Primer sequence: Antisense	Product size (bp)	Tm °
Integration primers[1]					
OCT3/4	endo	CCCCAGGGCCCCATTTTGGTACC	ACCTCAGTTTGAATGCATGGGAGAGC	143 bp	66
	pla	CATTCAAACTGAGGTAAGGG	TAGCGTAAAAGGAGCAACATAG	124 bp	55
LIN28	endo	AGCCATATGGTAGCCTCATGTCCGC	TCAATTCTGTGCCTCCGGGAGCAGGGTAGG	129 bp	66
	pla	AGCCATATGGTAGCCTCATGTCCGC	TAGCGTAAAAGGAGCAACATAG	251 bp	55
SOX2	endo	TTCACATGTCCCAGCACTACCAGA	TCACATGTGTGAGAGGGGCAGTGTGC	80 bp	62
	pla	TTCACATGTCCCAGCACTACCAGA	TTTGTTTGACAGGAGCGACAAT	111 bp	55
Differentiation primers					
GAPDH[2]		GAAGGTGAAGGTCGGAGTC	GAAGATGGTGATGGGATTTC	226 bp	60
OCT4[3]		GGGGGTTCTATTTGGGAAGGTA	ACCCACTTCTGCAGCAAGGG	111 bp	60
GFAP		AGGAGGAGGTTCGGGAACTC	CGCCATTGCCTCATACTGC	106bp	60
NKX6-1		GGGCCCGAGAGGGCTCGTTT	TCGTTCTCCGAGGCCCCCTTG	173bp	60
SOX1[4]		CCTGTGTGTACCCTGGAGTTTCTGT	TGCACGAAGCACCTGCAATAAGATG	174 bp	60
PAX6[4]		TCGAAGGGCCAAATGGAGAAGAGAAG	GGTGGGTTGTGGAATTGGTTGGTAGA	130 bp	60
PECAM1[5]		GAGTCCTGCTGACCCTTCTG	ATTTTGCACCGTCCAGTCC	69 bp	60
CD34[5]		TGGACCGCGCTTTGCT	CCCTGGGTAGGTAACTCTGGG	56 bp	60
ISL-1		TTGTACGGGATCAAATGCGCCAAG	AGGCCACACAGCGGAAACA	109 bp	60
TBX3[6]		GCTCTTCTCCCGTGCCTCTCTC	CCCTCCCTCTCTTTCCCTCTGTTC	135 bp	60
MYH6		AAGCTCAAGAACGCCTAC	CATTCTTTCCTCCTTCTCC	121 bp	60
SOX17[4]		AGGAAATCCTCAGACTCCTGGGTT	CCCAAACTGTTCAAGTGGCAGACA	111 bp	60
AFP		GCCAAGCTCAGGGTGTAG	CAATGACAGCCTCAAGTTGT	85 bp	60
ALB		GGTGTGTTTCGTCGAGATG	ACTGAGCAAAGGCAATCAAC	83 bp	60

Tm° indicates melting temperature in degrees Celsius.

endo; for detection of endogenous sequence, pla; for detection of plasmid vector-derived expression

Primer sequences were obtained from:

[1] Okita et al. (2011) Nature Methods, 8:409-412

[2] Qin et al. (2010) PLoS ONE, 5 (9) e13014

[3] Tran et al. (2009) Stem Cells 27 (8) 1869–1878

[4] Yang et al. (2008) Nature 453, 524–528

[5] Zambidis et al. (2005) Blood, 106(3): 860–870

[6] Calvanese et al.(2010) PNAS,107(31):13736–13741
